# Patient Characteristics and Real-World Use of Botulinum Toxins for the Treatment of Cervical Dystonia, Blepharospasm, and Hemifacial Spasm

**DOI:** 10.3390/toxins16080362

**Published:** 2024-08-16

**Authors:** Michael A. Hast, Amanda M. Kong, Shaina Desai, Soo Back, Sahar Syed, Jordan Holmes

**Affiliations:** 1 Merz Therapeutics, Raleigh, NC 27615, USA; jordan.holmes@merz.com; 2 Aetion, New York, NY 10001, USA; amanda.kong@aetion.com (A.M.K.); shaina.desai@aetion.com (S.D.); soowoo.back@gmail.com (S.B.); saharsyed.95@gmail.com (S.S.)

**Keywords:** botulinum toxin, cervical dystonia, blepharospasm, hemifacial spasm, treatment utilization, adverse events, movement disorders

## Abstract

Movement disorders such as cervical dystonia, blepharospasm, and hemifacial spasm negatively impact the quality of life of people living with these conditions. Botulinum toxin (BoNT) injections are commonly used to treat these disorders. We sought to describe patient characteristics, BoNT utilization, and potential adverse events (AEs) among patients with cervical dystonia, blepharospasm, and hemifacial spasm using Optum’s de-identified Clinformatics^®^ Data Mart Database. Patients were required to have a diagnosis of the specific condition plus evidence of treatment with BoNT between 8/1/2010 and 5/31/2022. Cervical dystonia patients were commonly females (76%) and aged 45 and older (78%); both blepharospasm and hemifacial spasm patients were commonly females (both 69%) and aged 65 and older (61% and 56%, respectively). Anticholinergics were commonly used (65–82% across cohorts), as were peripheral muscle relaxants for cervical dystonia patients specifically (31%). The median number of injections per year was 2 with the median weeks between injections being between 13 and 15. Of the AEs evaluated, dyspnea was identified frequently across all the cohorts (14–20%). The findings were similar for different BoNT formulations. More research is needed to thoroughly describe BoNT utilization, such as the doses injected, and to optimize treatment for patients with these conditions.

## 1. Introduction

Cervical dystonia (also known as spasmodic torticollis), blepharospasm, and hemifacial spasm are neurological conditions that can have a detrimental impact on the quality of life of patients living with them. Cervical dystonia is characterized by focal spasms in the neck and affects approximately 10 people per 100,000 [1]. Blepharospasm, or abnormal contractions of the muscles of the eyelids, affects approximately 3 people per 100,000 [1]. Hemifacial spasm involves muscle contractions of the portion of the face innervated by the seventh cranial nerve. The condition can be classified as primary or secondary, occurring due to nerve damage from another condition, including neoplasms, infections, structural abnormalities, and Bell’s palsy [2]. Hemifacial spasm affects approximately 10 people per 100,000 [3]. Studies have found that cervical dystonia results in decreases in the quality of life due to pain, reduced physical function, and the emotional impact of the condition [4,5]. Similarly, patients with blepharospasm and patients with hemifacial spasm have reported that these conditions impacted their activities of daily living, given the potentially profound effects on their vision, and left them feeling stigmatized and with appearance-related stress [6].

There are several treatment options to reduce the symptom burden experienced by patients, including anticholinergics and injections with botulinum toxins (BoNT). Therapies under development include cell therapy [7]. BoNT works by preventing the release of the neurotransmitter acetylcholine, producing paralysis in the targeted muscle and thereby reducing muscle contraction and spasms [8] and has been shown to increase patients’ quality of life [9,10]. There are currently five BoNTs approved by the United States (US) Food and Drug Administration (FDA) for cervical dystonia in adults—incobotulinumtoxinA (Xeomin) [11,12], onabotulinumtoxinA (Botox) [13,14], abobotulinumtoxinA (Dysport) [15,16,17], daxibotulinumtoxinA-lanm (Daxxify) [18,19], and rimabotulinumtoxinB [Myobloc] [20,21,22] and two for blepharospasm—incobotulinumtoxinA [23] and onabotulinumtoxinA [24]. There are no currently approved therapies for hemifacial spasm, although BoNT is routinely used off-label in clinical practice [25]. BoNT can also be used to treat facial asymmetry due to other types of palsy or synkinesis [26].

Despite the widespread use of BoNT for cervical dystonia, blepharospasm, and hemifacial spasm, there is limited research in real-world settings describing the characteristics of patients treated with these therapies or their treatment patterns. Therefore, the primary objectives of this study were to describe the characteristics of patients with cervical dystonia, blepharospasm, or hemifacial spasm treated with BoNT and describe treatment utilization using a large real-world data source. The secondary objective was to describe potential adverse events (AEs) occurring after BoNT injection. IncobotulinumtoxinA is unique in that it is purified, containing only the therapeutic neurotoxin component without the additional proteins contained in other formulations [27]; therefore, we present results stratified by incobotulinumtoxinA vs. other BoNT. This analysis was descriptive only, with the non-incobotulinumtoxinA results included to contextualize the incobotulinumtoxinA findings.

## 2. Results

### 2.1. Patient Populations

**Figure 1:**
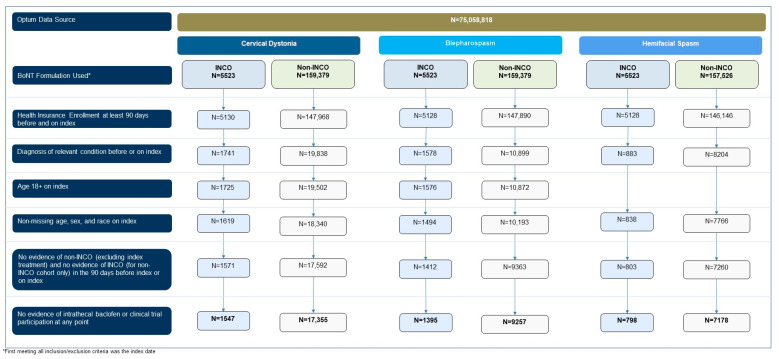
Patient attrition for cervical dystonia, blepharospasm, and hemifacial spasm patient populations.

We analyzed a total of 18,902 cervical dystonia patients (incobotulinumtoxinA users = 1547 and non-incobotulinumtoxinA users = 17,355), 10,652 blepharospasm patients (incobotulinumtoxinA users = 1395 and non-incobotulinumtoxinA users = 9257), and 7976 hemifacial spasm patients (incobotulinumtoxinA users = 798 and non-incobotulinumtoxinA users = 7718) (Figure 1).

The cervical dystonia patient population tended to be younger and had a larger proportion of females than the blepharospasm and hemifacial spasm patient populations. In all the patient populations, the incobotulinumtoxinA users tended to be older than the non-incobotulinumtoxinA users. Very few hemifacial spasm patients were less than 18 years old (Table 1). The proportion of patients who were non-Hispanic White was greater in the blepharospasm and hemifacial spasm patient populations compared to the cervical dystonia patients, and the incobotulinumtoxinA user cohort within the blepharospasm and hemifacial spasm patient populations tended to have more non-Hispanic White patients than the non-incobotulinumtoxinA user cohorts. The regional distributions of the three patient populations reflect that of the overall data source with the largest proportion of patients coming from the South. The proportions of incobotulinumtoxinA users from the South tended to be higher than the proportions of non-incobotulinumtoxinA users from the South in all the patient populations. Among the cervical dystonia patients, the use of anticholinergic drugs and peripheral muscle relaxants was common in both incobotulinumtoxinA users and non-incobotulinumtoxinA users in the baseline and follow-up periods. Anticholinergic drugs were also commonly used by blepharospasm and hemifacial spasms patients but to a lesser degree. Prior onabotulinumtoxinA use was recorded in more than one-third of patients but was more common among blepharospasm and hemifacial spasm patients.

### 2.2. BoNT Utilization

Utilization is reported among patients whose injection cadence was aligned with BoNT usage guidelines (those with no injections <4 weeks apart or >32 weeks apart; sensitivity analysis) and among all the patients. In the sensitivity analysis, there were 12,461 cervical dystonia patients (incobotulinumtoxinA users = 1218 and non-incobotulinumtoxinA users = 11,243), 6156 blepharospasm patients (incobotulinumtoxinA users = 1002 and non-incobotulinumtoxinA users = 5154), and 4540 hemifacial spasm patients (incobotulinumtoxinA users = 567 and non-incobotulinumtoxinA users = 3973) (Table 2). The median follow-up time was approximately 6 months (0.5 years) while the mean follow-up was closer to 1 year and tended to be longer among the non-incobotulinumtoxinA users in all three patient populations. The median number of injections per year was two and among people with more than one injection (approximately 60% of patients), the median number of weeks between injections was approximately 14, while the mean was approximately 15 weeks. Between 12 and 15% of incobotulinumtoxinA users switched to a different BoNT during follow-up, most commonly onabotulinumtoxinA. The observation of switching during follow-up was uncommon among non-incobotulinumtoxinA users; however, this is due in part to the hierarchical approach taken with patient selection where the patients who had ever used incobotulinumtoxinA were put into the incobotulinumtoxinA user cohort. Between 31% and 47% of incobotulinumtoxinA users had evidence of prior use of onabotulinumtoxinA, which indicates a switch from onabotulinumtoxinA to incobotulinumtoxinA (Table 1). The utilization results for the overall patient populations can be found in Table S1. When evaluating all the patients, the average time between injections tended to be longer and more patients were classified as having switched.

### 2.3. Potential AEs

**Figure 2:**
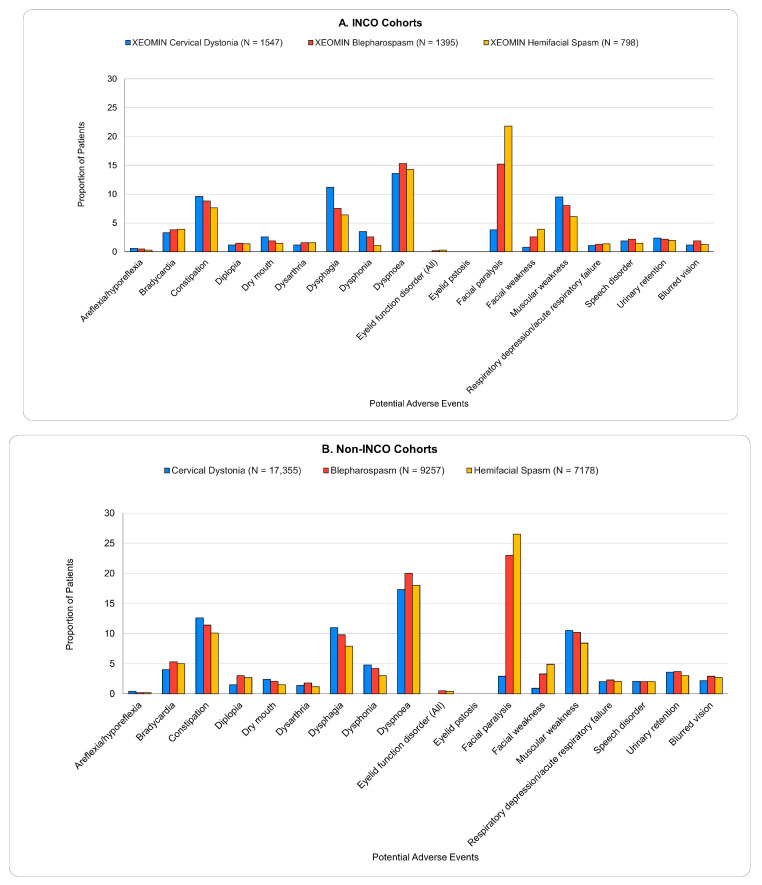
(**A**,**B**) Proportions of Patients Experiencing a Potential AE within 5 Months of Botulinum Toxin Administration.

The potential AEs evaluated in this study were those that were reported in incobotulinumtoxinA clinical trials—areflexia/hyporeflexia, bradycardia, constipation, diplopia, dry mouth, dysarthria, dysphagia, dysphonia, dyspnoea, eyelid function disorder (including eyelid ptosis), facial paralysis/paresis, muscular weakness, acute respiratory failure, speech disorder, urinary retention, and blurred vision—and were identified by diagnosis codes. Among the patients with cervical dystonia, blepharospasm, and hemifacial spasm, less than 12% of the patients had each AE of interest evaluated in this study during the 90 days prior to the index date. The most common conditions were dyspnea (which was coded in about 5% of the patients) and facial paralysis (which was coded in about 10–12% of the blepharospasm and hemifacial spasm patients) (Table S2). During the 5-month follow-up period, the most common potential AEs evaluated here were dyspnoea, dysphagia, muscular weakness, and constipation (>5%) among all three patient groups (Figure 2A,B). Among the patients with blepharospasm or hemifacial spasm, a diagnosis code for facial paralysis was also present in 15–27% of the patients, and among the non-incobotulinumtoxinA users with blepharospasm or hemifacial spasm, a diagnosis of bradycardia was present in approximately 5% of the patients. Overall, the prevalence of potential AEs was similar for different BoNT formulations. There was a difference of greater than five percentage points between the incobotulinumtoxinA users and the non-incobotulinumtoxinA users for only one AE—facial paralysis—among the blepharospasm patients. When evaluating AEs over a 1-month period following BoNT injection, the trends were similar in terms of the most common AEs experienced; however, with the shorter follow-up period, the number of patients experiencing the events was smaller than during the 5-month follow-up period (Figure S1).

## 3. Discussion

This claims-based study provides insight into the characteristics of insured patients with cervical dystonia, blepharospasm, or hemifacial spasm treated with a BoNT. The cervical dystonia patients were commonly non-Hispanic White females aged 45 years and older while the blepharospasm and hemifacial spasm patients were commonly non-Hispanic White female patients aged 65 years and older. The incobotulinumtoxinA user cohorts across the three populations tended to have higher proportions of patients from the South than the non-incobotulinumtoxinA user cohorts, which indicates potential regional differences in prescribing patterns. The prevalence of co-medications, such as anticholinergics, among all three patient populations and peripheral muscle relaxants among cervical dystonia patients suggests that the management of the symptoms of these conditions involves other therapies beyond BoNT. The use of multiple BoNT formulations was relatively common. In terms of treatment utilization, there were no major differences in the number of injections or weeks between injections between the different botulinum toxin formulations for the different conditions. The number of injections during follow-up (a median of approximately two injections) was consistent with the amount of follow-up for patients in this data source, which was less than 1 year for most patients; however, the time between injections (13–15 weeks across cohorts) aligned with the label-recommended injection intervals and published clinical trials [11,12,13,14,15,16,17,18,19,20,21,22,23,24].

There has been limited research published describing patients utilizing BoNT for these conditions in real-world data sources. Where research does exist, the studied population tends to be patients with cervical dystonia rather than blepharospasm or hemifacial spasm. A study of 1529 adult patients in the US treated with BoNT from 2016 to 2018 found that patients were on average 53 years old and 70% were female [28]. Approximately one-fifth of patients were treated with benzodiazepines and one-third were treated with muscle relaxants [28]. The average number of injections over a required 1-year follow-up period was 2.9 [28]. The most commonly used BoNT was onabotulinumtoxinA and most injections were performed by a neurologist [25]. Another real-world analysis of US patients using BoNT for different indications found that the average weeks between injections was approximately 15 for cervical dystonia and 16 for blepharospasm, irrespective of the BoNT formulation [29]. These additional published results are similar to those of the present analysis.

The most common potential AEs following BoNT injection identified in the populations of patients analyzed here were dyspnoea, dysphagia, muscular weakness, and constipation, plus facial paralysis for the blepharospasm and hemifacial spasm populations specifically. These are generally expected given the locations of injection and were observed in the published clinical trials [11,12,13,14,15,16,17,18,19,20,21,22,23,24]. Generally, most AEs evaluated here were uncommon. Only two AEs, facial paralysis and dyspnea, occurred in more than 15% of people in this study. Outside of the clinical trial setting, most information on BoNT AEs comes from spontaneous reporting systems such as the FDA’s Adverse Event Report System (FAERS) [30]. Regarding the use of BoNT across indications and for cosmetic purposes, the most common AEs (>2%) reported to the FAERS include drug ineffectiveness, dysphagia, and muscular weakness [31]. Information about AEs from epidemiological studies is limited [30]. A German study of 303 cervical dystonia patients receiving at least six BoNT injections between 1988 and 1995 found that AEs were reported after 22% of injections, with dysphagia being the most common, typically of low or moderate severity [32]. A Canadian analysis of 106 cervical dystonia patients, 70 hemifacial spasm patients, and 36 blepharospasm patients from 1990 to 1999 treated for over 2 years reported that 16% of cervical dystonia patients, 30% of hemifacial spasm patients, and 61% of blepharospasm patients experienced an AE at some point during their treatment [33]. The proportion of injections after which an AE was experienced ranged from 2% to 10% [33]. The most common AE for cervical dystonia was dysphagia and for hemifacial spasm and blepharospasm was eyelid ptosis [33]. A more recent study of blepharospasm patients from a German institution also reported eyelid ptosis as the most common AE [34]. Interestingly, eyelid ptosis was not found in this analysis; however, it is possible that providers may have coded it as facial paralysis.

There are several strengths of this research. First, this study was conducted on a large, geographically diverse data source of insured patients in the US. Second, while much of the data for these patient populations comes from clinical studies, real-world analyses such as this one reflect the actual patient populations treated with BoNT, including certain populations that may have been underrepresented in trials, and reflect utilization and treatment patterns in clinical practice. Third, this analysis adds to a body of work in which there is a limited number of studies using data collected recently using larger sample sizes. Blepharospasm and hemifacial spasms, in particular, are understudied. There are also limitations of this work that should be noted. Claims that the data were not collected for research purposes and errors in diagnostic coding may exist. The results described in this analysis may not be generalizable to other populations with different insurance coverage or uninsured patients. Regional variations in the use of BoNT may exist and sampling in CDM varies by state [35]. The race information was not based on self-report but rather on imputation conducted by a third-party vendor, and validated algorithms incorporating racial and ethnic neighborhood composition as ascertained by the US Census, residential zip code, and first and last name were used [36]. Among the Black participants, an imputed race variable using similar methods demonstrated moderate sensitivity (48%) and high specificity (97%) with a positive predictive value of 71% compared to self-reported race [36].

BoNT may have been administered for conditions other than the conditions analyzed as patients may have more than one condition for which BoNT is indicated. Switching results during follow-up must be viewed with the caveat that patients were hierarchically selected into the incobotulinumtoxinA cohort and, therefore, switching post-initiation may be under-estimated. Within this data, it is not possible to attribute the potential AEs definitively to BoNT injection. Certain potential AEs captured here may be associated with an underlying condition experienced by the patient. AEs may have been underreported if a patient did not seek medical care that generated a claim. There were a number of patients in the data that had unexpected patterns of use with a very short time between injections (<4 weeks) or a very long time (>32 weeks). It is unclear if these were due to coding errors or different muscles being injected at different times for other indications. To address this issue, the sensitivity analysis limiting the populations to patients with more standard utilization patterns was conducted. No statistical comparisons were made, and adjustments for confounding were applied; accordingly, any comparisons between patient populations or cohorts reported here were qualitative only. The specific muscles injected and the doses used were not available in this data source. Similarly, clinical information such as the type of hemifacial spasm, the extent of the severity for any of the three conditions, and a complete patient history were not available. Therefore, more research on other data sources, such as those with provider notes, is needed to more thoroughly describe treatment patterns among these patient populations.

## 4. Conclusions

This claims-based analysis provides insights into the characteristics, BoNT utilization patterns, and potential AEs of insured patients with cervical dystonia, blepharospasm, and hemifacial spasm treated with BoNTs in a real-world setting. The prevalence of co-medication use as well as the common usage of multiple BoNT formulations highlights the difficulties in treating these conditions, the symptoms of which can negatively impact the quality of life of those living with them. More research is needed to thoroughly describe BoNT utilization, such as the doses injected overall and in the context of monotherapy versus combination therapy, and optimize treatment for patients with dystonia and spasticity. Formal comparative studies between formulations adjusting for confounding may also be warranted.

## 5. Materials and Methods

### 5.1. Data Source

This study utilized Optum’s de-identified Clinformatics^®^ Data Mart Database (CDM). CDM is derived from a database of administrative health claims for members of large commercial and Medicare Advantage health plans. It utilizes medical and pharmacy claims to derive patient-level enrollment information, health care costs, and resource utilization information. The population is geographically diverse, spanning all 50 states and is statistically de-identified under the Expert Determination method consistent with HIPAA and managed according to Optum^®^ customer data use agreements. CDM administrative claims submitted for payment by providers and pharmacies are verified, adjudicated, and de-identified prior to inclusion. In this data, race/ethnicity is based on data collected from public records and by imputation that employs validated algorithms incorporating racial and ethnic neighborhood composition as ascertained by the US Census, residential zip code, and first and last name [36].

### 5.2. Study Design

An observational, retrospective cohort study was conducted. Patients entered the cohort on the date of a BoNT injection (index date). The potential index dates ranged from 1 August 2010 (availability of incobotulinumtoxinA following initial Food and Drug Administration approval) through 31 May 2022. The full study period ranged from 1 May 2010 through 30 June 2022 to allow for the 90-day baseline period and a minimum of 30 days of follow-up. The patients were followed from the index date until the earliest of the following: disenrollment in the health plan (allowing up to 45-day gaps), switch to another non-index botulinum toxin, end of data, or death.

### 5.3. Patient Selection

Three cohorts were created in the CDM data. The cohorts were not mutually exclusive so patients who had more than one of the conditions evaluated here could be included in more than one cohort. A hierarchy was applied to prioritize incobotulinumtoxinA use so that for the patients who had evidence of both incobotulinumtoxinA and non-incobotulinumtoxinA BoNT use, incobotulinumtoxinA was preferentially chosen as the index drug. To be included in the study, the patients were required to have a medical claim with a Healthcare Common Procedure Coding System (HCPCS) or a National Drug Code (NDC) for a BoNT (incobotulinumtoxinA, onabotulinumtoxinA, abobotulinumtoxinA, rimabotulinumtoxinB [cervical dystonia and blepharospasm only]) between 1 August 2011 and 31 May 2022 (date of BoNT injection was referred to as the index date and the specific BoNT injected was referred to as the index BoNT). DaxibotulinumtoxinA-lanm was not included in this analysis given its recent approval date. The patients were also required to have a medical claim with an International Classification of Disease, Ninth Edition, Clinical Modification (ICD-9-CM) or ICD-10-CM code for one of the three indications of interest prior to the index date or on the index date (cervical dystonia: ICD-9-CM: 723.5, 333.83; ICD-10-CM: M43.6, G24.3; blepharospasm: ICD-9-CM: 333.81; ICD-10-CM: G24.5; hemifacial spasm: ICD-9-CM: 351.8; ICD-10-CM: G51.3, G51.31, G51.32, G51.33, G51.36, G51.39) and have continuous enrollment (allowing up to 45-day gaps) 90 days prior to the index date through 30 days after the index date. The cervical dystonia and blepharospasm patients were required to be at least 18 years old on the index date, aligning with the product approvals. Patients were excluded from the cohort if they had any of the following: missing sex or race, a medical or pharmacy claim with an HCPCS or NDC for intrathecal baclofen at any time because of the potential to cause muscle weakness, a medical claim with an ICD-9-CM/ICD-10-CM diagnosis code indicating clinical trial participation (ICD-9-CM V70.7, ICD-10 Z00.6) at any time because their outcomes and care patterns may not have reflected typical patients, or evidence of more than one type of BoNT on the index date. IncobotulinumtoxinA users were excluded if they had evidence of administration of any non-incobotulinumtoxinA botulinum toxin in the 90 days prior to the index date or on the index date.

### 5.4. Patient Characteristics

The patient characteristics evaluated in this study included demographics, co-medications, and prior BoNT use. The patient demographics were measured on the index date and included age, sex, region, and race/ethnicity. To determine co-medication use, medical and pharmacy claims occurring 60 days prior to through 30 days after each injection, including the index date, were evaluated for the presence of procedure codes, NDCs, and generic names for the following medications: aminoglycoside antibiotics, parenterally administered drugs that interfere with neuromuscular transmission, aminoquinolines, anticholinergic drugs, anticoagulants, and peripheral muscle relaxants. Finally, all the medical and pharmacy claims prior to the index date were evaluated for prior BoNT use based on procedure codes, NDCs, and generic names.

### 5.5. Treatment Utilization

Treatment utilization was measured over follow-up based on medical and pharmacy claims for BoNT and was summarized using three utilization metrics: the number of days with a BoNT injection during follow-up, the average number of weeks between BoNT injections, and evidence of switching to a non-index BoNT based on a claim for a BoNT other than the patient’s index BoNT.

### 5.6. Potential Adverse Events

Potential AEs were defined by the presence of at least one medical claim in any setting with a diagnosis code for the potential AE of interest occurring up to 5 months (150 days) after each BoNT injection (inclusive) or until the next BoNT injection or censoring if it occurred earlier than 150 days. An assessment period of 5 months was chosen to account for the variability between botulinum toxin administration in clinical practice and the potential future availability of toxin treatments with purportedly longer efficacy, requiring less frequent injections. As all of the potential AEs were expected to be acute and resolved, there was no wash-out window (i.e., required time without evidence of AE). The following potential AEs were assessed: areflexia/hyporeflexia, bradycardia, constipation, diplopia, dry mouth, dysarthria, dysphagia, dysphonia, dyspnoea, eyelid function disorder (including eyelid ptosis), facial paralysis/paresis, muscular weakness, acute respiratory failure, speech disorder, urinary retention, and blurred vision. These AEs have been reported in incobotulinumtoxinA clinical trials [11,12,23,37,38,39]. In a sensitivity analysis, the time window for assessing potential AEs was shortened from 5 months to 1 month (30 days) as events occurring in closer proximity to the toxin injection were more likely to be related to the procedure.

### 5.7. Statistical Analyses

This analysis was not comparative. The three patient populations were analyzed separately. A single patient could be in more than one population if the individual had more than one condition of interest. The results were stratified by the use of incobotulinumtoxinA vs. a non-incobotulinumtoxinA BoNT on the index date. The similarities and differences between the incobotulinumtoxinA and non-incobotulinumtoxinA cohorts were described qualitatively, but no statistical tests to determine significance were performed.

The continuous patient characteristics were described by the mean, standard deviation (SD), median, and interquartile range (IQR). The categorical patient characteristics were described as the count and percentage. The utilization outcomes were analyzed in the following ways. The number of injections was summarized as a rate, calculated as the number of injections divided by person-time, and expressed as the number of injections per year at the patient level (e.g., Patient A had four injections per year during follow-up). The mean (SD) and median (IQR) number of injections per patient per year were presented at the cohort level (e.g., on average, men had 3 injections per year). The average time between injections was summarized as the mean number of weeks between injections at the patient level (e.g., Patient A had an average of 15 weeks between injections) and aggregated to present the mean (SD) and median (IQR) average number of months between injections at the cohort level (e.g., on average, men had a mean of 20 weeks between injections). Switching was described as the count and proportions of patients who had evidence of any non-index BoNT during follow-up. The specific type of non-index treatment first switched to after the index treatment was reported as counts and proportions. The count of patients and proportion of the cohort experiencing each potential AE during follow-up was reported, which is consistent with the Clinical Study Reports from the incobotulinumtoxinA clinical trials.

A sensitivity analysis was conducted limiting all the cohorts to patients with no BoNT injections less than 4 weeks or greater than 32 weeks apart. The purpose of this sensitivity analysis was to evaluate the utilization metrics with an injection cadence more aligned with the guidelines of BoNT usage. In this analysis, an additional criterion was added to determine the end of follow-up: 16 weeks after the final injection before a >32-week gap.

The analyses were conducted using the Aetion Evidence Platform (Aetion, Inc., New York, NY, USA).

## Figures and Tables

**Table 1:** Patient demographic characteristics, co-medication use, and prior botulinum toxin use.

	Cervical Dystonia		Blepharospasm		Hemifacial Spasm	
	INCO	Non-INCO Botulinum Toxin	INCO	Non-INCO Botulinum Toxin	INCO	Non-INCO Botulinum Toxin
	N = 1547	N = 17,355	N = 1395	N = 9257	N = 798	N = 7178
**Age**						
Mean, SD	58.83 (14.88)	56.80 (15.18)	67.36 (12.47)	65.14 (13.56)	66.66 (13.19)	63.27 (14.48)
Median, IQR	60.00 [48.00, 70.00]	57.00 [46.00, 68.00]	69.00 [61.00, 76.00]	67.00 [57.00, 75.00]	68.00 [59.00, 76.00]	66.00 [54.00, 74.00]
<18 years (N, %)	-	-	-	-	1 (0.1%)	27 (0.4%)
18–44 years (N, %)	284 (18.4%)	3868 (22.3%)	82 (5.9%)	802 (8.7%)	55 (6.9%)	811 (11.3%)
45–64 years (N, %)	650 (42.0%)	7559 (43.6%)	374 (26.8%)	2882 (31.1%)	216 (27.1%)	2389 (33.3%)
65+ years (N, %)	613 (39.6%)	5928 (34.2%)	939 (67.3%)	5573 (60.2%)	526 (65.9%)	3951 (55.0%)
**Sex (N, %)**						
Male	380 (24.6%)	4124 (23.8%)	444 (31.8%)	2811 (30.4%)	247 (31.0%)	2186 (30.5%)
Female	1167 (75.4%)	13,231 (76.2%)	951 (68.2%)	6446 (69.6%)	551 (69.0%)	4992 (69.5%)
**Race (N, %)**						
Non-Hispanic White	1301 (84.1%)	14,430 (83.1%)	1009 (72.3%)	6878 (74.3%)	532 (66.7%)	5076 (70.7%)
Non-Hispanic Black	111 (7.2%)	1185 (6.8%)	121 (8.7%)	703 (7.6%)	73 (9.1%)	542 (7.6%)
Non-Hispanic Asian	42 (2.7%)	381 (2.2%)	61 (4.4%)	507 (5.5%)	59 (7.4%)	483 (6.7%)
Hispanic	93 (6.0%)	1359 (7.8%)	204 (14.6%)	1169 (12.6%)	134 (16.8%)	1077 (15.0%)
Other	0 (0.0%)	0 (0.0%)	0 (0.0%)	0 (0.0%)	0 (0.0%)	0 (0.0%)
**Region (N, %)**						
Northeast	129 (8.3%)	1981 (11.4%)	130 (9.3%)	1095 (11.8%)	79 (9.9%)	893 (12.4%)
Midwest	347 (22.4%)	4069 (23.4%)	283 (20.3%)	2095 (22.6%)	181 (22.7%)	1802 (25.1%)
South	707 (45.7%)	7096 (40.9%)	693 (49.7%)	3851 (41.6%)	352 (44.1%)	2718 (37.9%)
West	363 (23.5%)	4193 (24.2%)	289 (20.7%)	2209 (23.9%)	185 (23.2%)	1753 (24.4%)
Other	0 (0.0%)	0 (0.0%)	0 (0.0%)	0 (0.0%)	0 (0.0%)	1 (0.0%)
Unknown	1 (0.1%)	16 (0.1%)	0 (0.0%)	7 (0.1%)	1 (0.1%)	11 (0.2%)
**Co-Medications at Index Date (60 Days Before to 30 Days After) (N, %)**						
Aminoglycoside antibiotics and spectinomycin	2 (0.1%)	56 (0.3%)	3 (0.2%)	20 (0.2%)	3 (0.4%)	13 (0.2%)
Parenterally administered drugs that interfere with neuromuscular transmission	5 (0.3%)	90 (0.5%)	3 (0.2%)	33 (0.4%)	2 (0.3%)	36 (0.5%)
Aminoquinolines	25 (1.6%)	264 (1.5%)	20 (1.4%)	86 (0.9%)	3 (0.4%)	76 (1.1%)
Anticholinergic drugs	1228 (79.4%)	14,207 (81.9%)	936 (67.1%)	6331 (68.4%)	516 (64.7%)	4771 (66.5%)
Anticoagulants	69 (4.5%)	865 (5.0%)	63 (4.5%)	495 (5.3%)	47 (5.9%)	352 (4.9%)
Peripheral muscle relaxants	453 (29.3%)	5326 (30.7%)	113 (8.1%)	850 (9.2%)	75 (9.4%)	692 (9.6%)
Co-Medications after Index Date (N, %) *						
Aminoglycoside antibiotics and spectinomycin	10 (0.6%)	147 (0.8%)	8 (0.6%)	81 (0.9%)	7 (0.9%)	53 (0.7%)
Parenterally administered drugs that interfere with neuromuscular transmission	12 (0.8%)	261 (1.5%)	15 (1.1%)	119 (1.3%)	2 (0.3%)	82 (1.1%)
Aminoquinolines	27 (1.7%)	276 (1.6%)	17 (1.2%)	118 (1.3%)	1 (0.1%)	90 (1.3%)
Anticholinergic drugs	861 (55.7%)	10,444 (60.2%)	662 (47.5%)	5539 (59.8%)	397 (49.7%)	4095 (57.0%)
Anticoagulants	76 (4.9%)	1068 (6.2%)	74 (5.3%)	797 (8.6%)	44 (5.5%)	571 (8.0%)
Peripheral muscle relaxants	381 (24.6%)	4661 (26.9%)	124 (8.9%)	1168 (12.6%)	69 (8.6%)	856 (11.9%)
**Previous Botulinum Toxin Use Any Time Prior to Index Date (N, %)**						
INCO	405 (26.2%)	0 (0.0%)	380 (27.2%)	0 (0.0%)	170 (21.3%)	0 (0.0%)
OnabotulinumtoxinA	486 (31.4%)	6151 (35.4%)	613 (43.9%)	4015 (43.4%)	376 (47.1%)	2953 (41.1%)
RimabotulinumtoxinB	51 (3.3%)	307 (1.8%)	16 (1.1%)	48 (0.5%)	-	-
AbobotulinumtoxinA	32 (2.1%)	184 (1.1%)	8 (0.6%)	40 (0.4%)	8 (1.0%)	26 (0.4%)
Any	772 (49.9%)	6483 (37.4%)	850 (60.9%)	4058 (43.8%)	477 (59.8%)	2965 (41.3%)

INCO, incobotulinumtoxinA; IQR, interquartile range; SD, standard deviation; * Co-medication occurring in the 60 days prior to 30 days after an injection. Percentages calculated among patients who had more than 1 injection (Cervical Dystonia INCO: n = 999; Cervical Dystonia Non-INCO: n = 11,819; Blepharospasm INCO: n = 871; Blepharospasm Non-INCO: n = 6762; Hemifacial Spasm INCO: n = 521; Hemifacial Spasm Non-INCO: n = 5169).

**Table 2:** Utilization of BoNT, Sensitivity Analysis Excluding Patients with BoNT Administrations <4 Weeks or >32 Weeks Apart.

	Cervical Dystonia		Blepharospasm		Hemifacial Spasm	
	INCO	Non-INCO Botulinum Toxin	INCO	Non-INCO Botulinum Toxin	INCO	Non-INCO Botulinum Toxin
	N = 1218	N = 11,243	N = 1002	N = 5154	N = 567	N = 3973
**Follow-Up, Person-Years**						
Mean per person, SD	0.85 (0.98)	0.94 (1.23)	0.88 (1.03)	1.23 (1.64)	0.91 (1.03)	1.15 (1.54)
Median, IQR	0.53 [0.31, 1.04]	0.51 [0.31, 1.04]	0.49 [0.31, 1.02]	0.57 [0.31, 1.47]	0.54 [0.31, 1.10]	0.55 [0.31, 1.33]
**Number of Injections**						
Mean number of injections per person, SD	3.28 (3.66)	3.56 (4.61)	3.35 (3.90)	4.54 (6.16)	3.35 (3.76)	4.16 (5.57)
Median, IQR	2.00 [1.00, 4.00]	2.00 [1.00, 4.00]	2.00 [1.00, 4.00]	2.00 [1.00, 5.00]	2.00 [1.00, 4.00]	2.00 [1.00, 5.00]
Mean number of injections per person-year, SD	2.24 (1.25)	2.18 (1.22)	2.17 (1.29)	2.27 (1.27)	2.17 (1.20)	2.20 (1.20)
Median, IQR	2.00 [1.00, 3.30]	2.00 [1.00, 3.16]	2.00 [1.00, 3.01]	2.00 [1.00, 3.34]	2.00 [1.00, 3.14]	2.00 [1.00, 3.23]
**Weeks between Injections**						
Number of patients with more than 1 injection	708 (58.1%)	6424 (57.1%)	554 (55.3%)	3055 (59.3%)	326 (57.5%)	2316 (58.3%)
Mean number of average weeks between injections per person, SD	14.51 (3.10)	14.73 (3.45)	15.13 (4.01)	15.41 (4.08)	15.64 (3.92)	15.65 (3.94)
Median, IQR	13.81 [13.00, 15.60]	13.86 [13.00, 15.86]	14.14 [13.00, 16.50]	14.44 [13.06, 17.07]	14.43 [13.25, 16.78]	14.64 [13.19, 17.23]
Switching * (N, %)						
Number of patients with an administration of a non-incobotulinumtoxinA BoNT during follow-up	147 (12.1%)	142 (1.3%)	143 (14.3%)	36 (0.7%)	83 (14.6%)	10 (0.3%)
OnabotulinumtoxinA use	131 (10.8%)		135 (13.5%)		81 (14.3%)	
RimabotulinumtoxinB use	4 (0.3%)		2 (0.2%)			
AbobotulinumtoxinA use	12 (1.0%)		6 (0.6%)		2 (0.4%)	
OnabotulinumtoxinA on index date and switch to AbobotulinumtoxinA		50 (0.4%)		12 (0.2%)		7 (0.2%)
OnabotulinumtoxinA on index date and switch to RimabotulinumtoxinB		32 (0.3%)		10 (0.2%)		
AbobotulinumtoxinA on index date and switch to OnabotulinumtoxinA		25 (0.2%)		7 (0.1%)		3 (0.1%)
AbobotulinumtoxinA on index date and switch to RimabotulinumtoxinB		1 (0.0%)		0 (0.0%)		
RimabotulinumtoxinB on index date and switch to OnabotulinumtoxinA		29 (0.3%)		7 (0.1%)		
RimabotulinumtoxinB on index date and switch to AbobotulinumtoxinA		5 (0.0%)		0 (0.0%)		

* First switch captured.

## Data Availability

Restrictions apply to the availability of these data. The data were obtained from Optum. For information on obtaining the underlying data used for this analysis, please contact Optum.

## Supplementary Materials

The following supporting information can be downloaded at: https://www.mdpi.com/article/10.3390/toxins16080362/s1, Table S1: Utilization of Botulinum Toxin, All Patients, Table S2: Evidence of Potential AEs During 90-Day Baseline Period, Figure S1: Proportions of Patients Experiencing a Potential AE within 1 Month of Botulinum Toxin Administration.
