# Supplementary material for: Patient Characteristics and Real-World Use of Botulinum Toxins for the Treatment of Cervical Dystonia, Blepharospasm, and Hemifacial Spasm
Source: Toxins (Basel). 2024 Aug 16;16(8):362. doi: 10.3390/toxins16080362 (PMC11359877; doi:10.3390/toxins16080362)
Supplement: Supplementary file 1 [file toxins-16-00362-s001.zip › toxins-3113926-supplementary.pdf]

Patient Characteristics and Real-World Use of Botulinum Toxins for the Treatment of Cervical Dystonia, Blepharospasm, and Hemifacial Spasm

Michael A. Hast, Amanda M. Kong, Shaina Desai, Soo Back, Sahar Syed and Jordan Holmes

Table S1. Utilization of Botulinum Toxin, All Patients.

	Cervical Dystonia		Blepharospasm		Hemifacial Spasm	
	**INCO**	**Non-INCO Botulinum Toxin**	**INCO**	**Non-INCO Botulinum Toxin**	**INCO**	**Non-INCO Botulinum Toxin**
	**N=1547**	**N=17,355**	**N=1395**	**N=9257**	**N=798**	**N=7178**
**Follow-Up, Person-Years**						
...Mean per person, SD	1.82 (1.96)	2.51 (2.43)	1.81 (1.89)	3.12 (2.80)	1.88 (1.88)	3.05 (2.77)
......Median, IQR	1.10 [0.48, 2.43]	1.68 [0.70, 3.56]	1.07 [0.50, 2.54]	2.32 [0.89, 4.55]	1.21 [0.55, 2.64]	2.22 [0.88, 4.43]
**Number of Injections**						
...Mean number of injections per person, SD	3.87 (4.47)	4.74 (5.83)	4.01 (5.43)	6.02 (7.25)	3.84 (4.33)	5.64 (6.64)
......Median, IQR	2.00 [1.00, 5.00]	3.00 [1.00, 6.00]	2.00 [1.00, 5.00]	3.00 [1.00, 8.00]	2.00 [1.00, 5.00]	3.00 [1.00, 7.00]
...Mean number of injections per person-year, SD	2.07 (1.42)	1.98 (1.45)	2.03 (1.44)	2.03 (1.46)	1.94 (1.23)	1.93 (1.34)
......Median, IQR	1.92 [1.00, 3.03]	1.63 [0.94, 3.00]	1.89 [1.00, 3.00]	1.85 [1.00, 3.00]	1.90 [1.00, 3.00]	1.73 [0.98, 3.00]
**Weeks between Injections**						
...N of patients with more than 1 injection	999 (64.6%)	11,819 (68.1%)	871 (62.4%)	6,762 (73.0%)	521 (65.3%)	5,169 (72.0%)
...Mean number of average weeks between	16.12 (12.14)	19.20 (19.01)	18.64 (15.48)	22.59 (22.49)	19.42 (13.08)	22.85 (22.14)

injections per person, SD						
......Median, IQR	13.86 [12.71, 16.33]	14.29 [12.77, 18.65]	14.43 [13.00, 19.00]	16.21 [13.24, 23.72]	15.07 [13.32, 20.79]	16.64 [13.44, 24.14]
**Switching* (N, %)**						
...Number of patients with an administration of a non-incobotulinumtoxinA BoNT during follow-up	354 (22.9%)	433 (2.5%)	426 (30.5%)	122 (1.3%)	238 (29.8%)	52 (0.7%)
......OnabotulinumtoxinA use	304 (19.7%)		407 (29.2%)		235 (29.4%)	
...... RimabotulinumtoxinB use	26 (1.7%)		9 (0.6%)			
...... AbobotulinumtoxinA use	24 (1.6%)		10 (0.7%)		3 (0.4%)	
...... OnabotulinumtoxinA on Index Date and Switch to AbobotulinumtoxinA		144 (0.8%)		60 (0.6%)		43 (0.6%)
...... OnabotulinumtoxinA on Index Date and Switch to RimabotulinumtoxinB		109 (0.6%)		29 (0.3%)		
...... AbobotulinumtoxinA on Index Date and Switch to OnabotulinumtoxinA		78 (0.4%)		15 (0.2%)		9 (0.1%)
...... AbobotulinumtoxinA on Index Date and Switch to		8 (0.0%)		1 (0.0%)		

Rimabotulinumtoxin B						
...... Rimabotulinumtoxin B on Index Date and Switch to Onabotulinumtoxin A	85 (0.5%)	18 (0.2%)				
...... Rimabotulinumtoxin B on Index Date and Switch to Abobotulinumtoxin A	10 (0.1%)	0 (0.0%)				

BoNT, botulinum toxin; INCO, incobotulinumtoxinA; IQR, interquartile range; SD, standard deviation. *First switch captured.

Table S2. Evidence of Potential AEs During 90-Day Baseline Period.

	**Cervical Dystonia**		**Blepharospasm**		**Hemifacial Spasm**	
	**INCO**	**Non-INCO Botulinum Toxin**	**INCO**	**Non-INCO Botulinum Toxin**	**INCO**	**Non-INCO Botulinum Toxin**
	**N=1547**	**N=17,355**	**N=1395**	**N=9257**	**N =798**	**N =7178**
**Potential Adverse Events at Baseline (90 Days to 1 Day Before Index Date) (N, %)**						
...Areflexia/hyporeflexia	5 (0.3%)	37 (0.2%)	2 (0.1%)	7 (0.1%)	0 (0.0%)	13 (0.2%)
...Bradycardia	7 (0.5%)	213 (1.2%)	22 (1.6%)	109 (1.2%)	17 (2.1%)	89 (1.2%)
...Constipation	54 (3.5%)	745 (4.3%)	39 (2.8%)	246 (2.7%)	20 (2.5%)	172 (2.4%)
...Diplopia	13 (0.8%)	110 (0.6%)	11 (0.8%)	78 (0.8%)	3 (0.4%)	64 (0.9%)
...Dry mouth	12 (0.8%)	118 (0.7%)	6 (0.4%)	60 (0.6%)	3 (0.4%)	37 (0.5%)
...Dysarthria	5 (0.3%)	117 (0.7%)	10 (0.7%)	50 (0.5%)	6 (0.8%)	25 (0.3%)
...Dysphagia	62 (4.0%)	792 (4.6%)	44 (3.2%)	267 (2.9%)	18 (2.3%)	181 (2.5%)
...Dysphonia	27 (1.7%)	317 (1.8%)	13 (0.9%)	109 (1.2%)	6 (0.8%)	68 (0.9%)

...Dyspnoea	73 (4.7%)	968 (5.6%)	87 (6.2%)	484 (5.2%)	40 (5.0%)	343 (4.8%)
...Eyelid function disorder (including eyelid ptosis)	0 (0.0%)	0 (0.0%)	0 (0.0%)	11 (0.1%)	2 (0.3%)	12 (0.2%)
......Eyelid pstosis	0 (0.0%)	0 (0.0%)	0 (0.0%)	0 (0.0%)	0 (0.0%)	0 (0.0%)
...Facial paralysis/paresis (paralysis)	31 (2.0%)	189 (1.1%)	134 (9.6%)	991 (10.7%)	92 (11.5%)	846 (11.8%)
...Facial paralysis/paresis (facial weakness)	6 (0.4%)	36 (0.2%)	5 (0.4%)	71 (0.8%)	4 (0.5%)	109 (1.5%)
...Muscular weakness	57 (3.7%)	757 (4.4%)	33 (2.4%)	238 (2.6%)	11 (1.4%)	164 (2.3%)
...Respiratory depression/acute respiratory failure	4 (0.3%)	75 (0.4%)	4 (0.3%)	36 (0.4%)	2 (0.3%)	24 (0.3%)
...Speech disorder	8 (0.5%)	122 (0.7%)	7 (0.5%)	51 (0.6%)	4 (0.5%)	44 (0.6%)
...Urinary retention	15 (1.0%)	227 (1.3%)	10 (0.7%)	91 (1.0%)	0 (0.0%)	48 (0.7%)
...Blurred vision	12 (0.8%)	139 (0.8%)	11 (0.8%)	76 (0.8%)	4 (0.5%)	56 (0.8%)

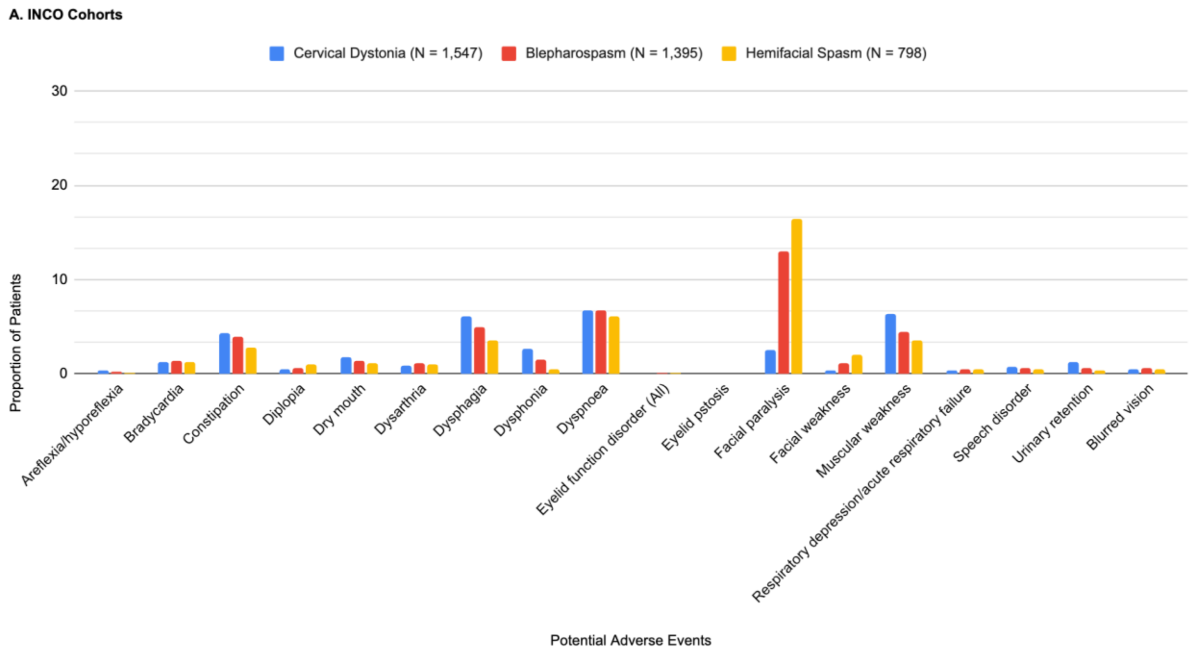

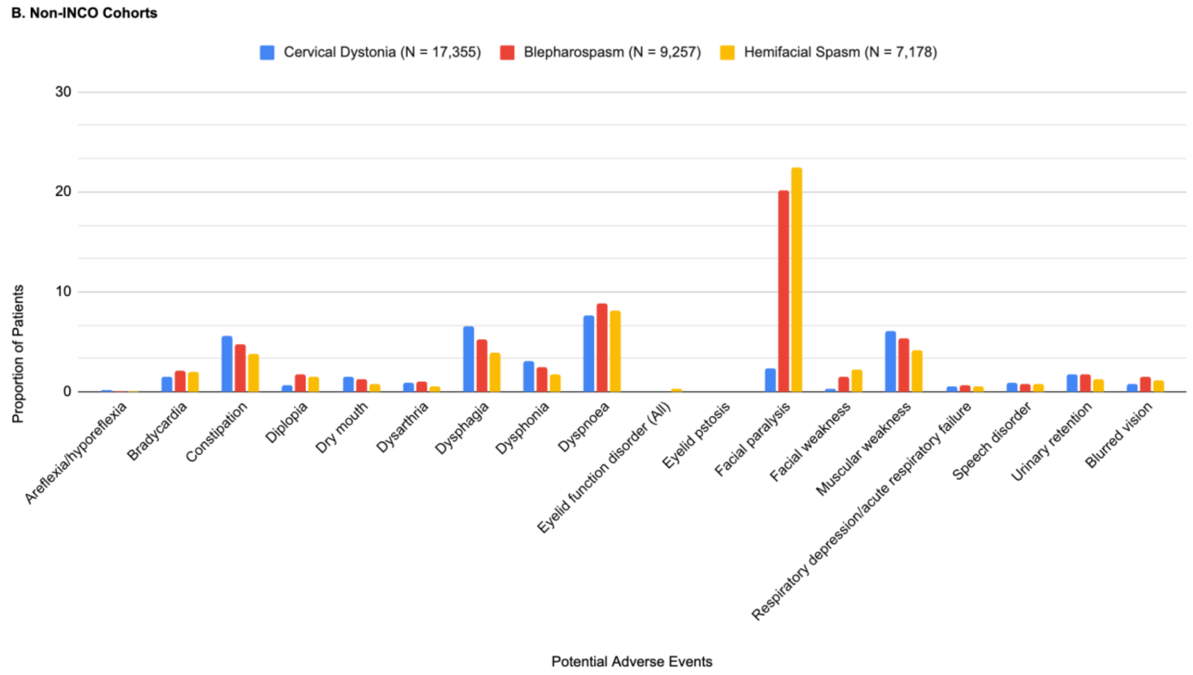

Figure S1. (**A**,**B**) Proportions of Patients Experiencing a Potential AE within 1 Month of Botulinum Toxin Administration.
